# CO-DESIGNING A MOBILE-BASED COGNITIVE TRAINING WITH OLDER CHINESE AMERICANS AND THEIR ADULT CHILDREN

**DOI:** 10.1093/geroni/igae098.0781

**Published:** 2024-12-31

**Authors:** Tingzhong (Michelle) Xue, Eleanor McConnell, Amy Wei, Bei Wu, Hanzhang Xu

**Affiliations:** Duke University School of Nursing, Durham, North Carolina, United States; Duke University, Durham, North Carolina, United States; Duke University School of Medicine, Durham, North Carolina, United States; New York University, New York, New York, United States; Duke University, Durham, North Carolina, United States

## Abstract

Older Chinese Americans are at high risk for dementia, yet often fail to access to prevention services/programs due to language barriers, transportation issues etc. BrainHQ is a mobile-based, effective cognitive training program that can potentially address these barriers and delay cognitive decline in older Chinese Americans. In this study, we will apply an experience-based co-design approach that leverages the existing BrainHQ features and older Chinese Americans’ prior knowledge, lived experience, and social norms around dementia to co-develop a cognitive training intervention. We first have worked with BrainHQ to translate and adapt more than 30 evidence-based cognitive training exercises into Chinese. We then hosted focus groups among older Chinese Americans (n=21) to understand their perceived benefits and barriers regarding participating in cognitive training. We also interviewed their adult children separately as they are often influenced by the ideology of filial piety and assume a major responsibility for caregiving. Utilizing the findings of the focus groups, we will then conduct an experience-based co-design workshop with older Chinese Americans (n=6), their adult children (n=6), and community stakeholders (n=2) to improve the cultural and linguistic relevance of the intervention. We will start with a journey map where participants are guided to brainstorm challenges (e.g. fears and anxieties related to performance, time constrains, distrust) they may experience when participating in the intervention. Then they will be asked to create prototypes of intervention components to address these challenges. These findings will facilitate better delivery of the cognitive training among older Chinese Americans and inform broader implementation.

